# The Timing of Intrauterine Exposure to Maternal SARS-CoV-2 Infection Impacts Neurodevelopment and Growth Trajectories During the First Year of Life

**DOI:** 10.3390/jcm15020600

**Published:** 2026-01-12

**Authors:** Thomas N. Griffin, Andrés M. Treviño-Alvarez, Tomás Cabeza de Baca, Paolo Piaggi, Asmaa Yehia, Beatriz E. Chávez-Luévanos, Osama A. Abulseoud

**Affiliations:** 1 Alix School of Medicine at Mayo Clinic, Phoenix, AZ 85054, USA; 2 Department of Psychiatry and Psychology, Mayo Clinic Arizona, Phoenix, AZ 85054, USA; trevinoalvarez.andres@mayo.edu; 3 Department of Neurology and Pediatric Neurology, Universidad Autonoma de Nuevo Leon, Monterrey 64460, Mexico; 4 Obesity & Diabetes Clinical Research Section, Phoenix Epidemiology & Clinical Research Branch, National Institute on Diabetes and Digestive and Kidney Diseases, National Institute of Health, Phoenix, AZ 85016, USA; tommy.cabezadebaca@nih.gov (T.C.d.B.);; 5 Department of Neuroscience, Graduate School of Biomedical Sciences, Mayo Clinic College of Medicine, Phoenix, AZ 85259, USA; hatem.asmaa@mayo.edu; 6 Department of Medical Physiology, Faculty of Medicine, Mansoura University, Mansoura 35516, Egypt

**Keywords:** maternal SARS-CoV-2, intrauterine exposure, neurodevelopment, growth trajectories

## Abstract

**Background:** The effect of intrauterine exposure to SARS-CoV-2 infection during pregnancy on neurodevelopment and growth trajectories during the first year of life remains under investigation. **Methods:** We retrospectively reviewed the electronic medical records of all pregnant women who received care at Mayo Health System and tested positive for SARS-CoV-2 (RT-PCR) from March 2020 through October 2021 and examined the effects of fetal sex and trimester of maternal SARS-CoV-2 infection on the risk of neurodevelopmental disorder diagnosis and growth trajectories of head circumference (HC) and body weight (BW) percentiles over the first year of life using linear mixed models. **Results:** We observed that a higher percentage of male infants (n = 357), compared to females (n = 344), have neurodevelopmental disorders (10.9% vs. 5.2%, *p* = 0.008), and infants exposed to maternal SARS-CoV-2 infection in the second (n = 183) or third trimester (n = 358) have a higher prevalence of neurological diagnoses compared to those exposed in the first trimester (n = 160) (1st vs. 2nd vs. 3rd trimester: 0% vs. 0.9% vs. 0.7%, respectively, *p* = 0.037). In addition, female infants, compared to males, had significantly lower BW (B = −0.04, *p* < 0.0001) and HC (B = −0.06, *p* < 0.0001) percentile growth trajectories over the first year of life. Moreover, infants exposed to maternal SARS-CoV-2 infection in the second trimester had a significantly lower BW percentile growth trajectory (B = −0.01, *p* = 0.006), while infants exposed to maternal SARS-CoV-2 infection in the third trimester had a significantly lower HC percentile growth trajectory (B = −0.02, *p* = 0.02). **Conclusions:** In utero exposure to maternal SARS-CoV-2 infection could have long-term effects on growth trajectories, depending on the infant’s sex and timing of exposure.

## 1. Introduction

Intrauterine exposure to maternal viral infections or being born during the stress of a pandemic increases the risk for neurodevelopmental disorders and other health problems. Several large-scale studies on birth cohorts from the 1918 influenza pandemic and the 1969–1970 Hong Kong flu pandemic document that fetal exposure to influenza during the pandemic is associated with reduced heights, lower intelligence scores, and less educational achievement [1,2,3,4]. The association between intrauterine exposure to maternal SARS-CoV-2 infection and the risk of neurodevelopmental disorders later in life remains under intense investigation [5,6,7,8]. Few studies report higher rates of developmental delay [9,10,11,12], while others found no difference [13,14,15,16] or even lower rates of positive autism screenings among prenatal SARS-CoV-2 exposure compared to unexposed children [17]. In addition, comparing children born during the pandemic, regardless of the exposure status, with pre-pandemic historic cohorts showed worse performance in several neurodevelopmental domains in one study [13] and no difference in the risk of autism in another study [17].

This wide variability is also evident in the studies that examined the effect of exposure to SARS-CoV-2 during pregnancy on growth trajectories. Ockene et al. reported a lower body mass index z-score at birth but a greater weight gain trajectory over the first year in SARS-CoV-2-exposed (n = 149) compared to unexposed (n = 127) infants [18]. One study from Uganda reported that SARS-CoV-2 infection in early pregnancy was associated with a lower median length z-score at age 3 months compared with no infection or late pregnancy infection [19]. In contrast, a large retrospective study found no differences in weight, length, or head circumference curves between pandemic infants born to SARS-CoV-2 positive (n = 758) and negative mothers (n = 9345) over the first 2 years of life. However, compared to pre-pandemic infants (n = 3221), the length of pandemic SARS-CoV-2 negative infants (n = 9345) was lower from birth to 9 months [20].

Some of this evident heterogeneity could be explained by differences in the time of infection during brain development [21]. In Ayad et al.’s study, the rate of neurodevelopmental delay among maternal SARS-CoV-2-exposed infants (n = 298) was higher among infants who were exposed during the first and second trimester compared to the third trimester [22]. In contrast, Edlow et al. reported that third-trimester infection was associated with a greater rate of neurodevelopmental diagnoses in a cohort of 222 infants born to SARS-CoV-2-positive mothers [23]. Firestein et al., on the other hand, found no association between the trimester of maternal infection and neurodevelopmental measures in a group of 112 infants with documented SARS-CoV-2 exposure and 37 infants with unknown timing of exposure or pregnancy compared to 258 infants with no documented exposure [24]. Similarly, Shuffrey et al. found no trimester effect on the risk for neurodevelopmental disorders in a group of 114 exposed compared to 141 unexposed infants [13] and Jaswa et al.’s study on 1757 infants did not identify increased risk based on trimester of infection [25].

Fetal sex is another variable that could influence neonatal outcome because perturbations to brain development under the stress of maternal infection differ between males and females [26,27,28]. Several animal studies provide compelling evidence of sex-dependent effects of SARS-CoV-2 viral or spike protein exposure in utero, with male offspring showing worse performance in different behavioral paradigms and more structural and molecular brain changes than females [29,30,31,32,33]. All these findings are to be considered in the light of the reported effect of COVID-19 on the nervous system. COVID-19 is associated with a broad spectrum of neurological manifestations, including anosmia, encephalopathy, stroke, seizures, and long-term cognitive and autonomic dysfunction [34]. Neurological injury is suggested to be driven primarily by indirect mechanisms rather than widespread neuronal infection. Despite not being fully identified, these mechanisms include neuroinflammation, endothelial and blood–brain barrier dysfunction, microvascular injury, immune-mediated processes, and metabolic stress affecting neural and glial cells [35].

In this study, we examined the effects of fetal sex and trimester of maternal SARS-CoV-2 asymptomatic and mildly symptomatic infection on the risk of neurodevelopmental disorder diagnosis, growth trajectories of head circumference and body weight percentiles over the first year of life.

## 2. Methods

### 2.1. Data Collection

This study was approved by the Mayo Clinic Institutional Review Board (ID: 21–010,940). It included all infants born to pregnant women who received medical care in the Mayo Clinic Health System (MCHS) from 1 March 2020, through 1 October 2021, with confirmed positive SARS-CoV-2 by reverse-transcriptase polymerase chain reaction (RT-PCR) test (nasopharyngeal/oropharyngeal swab specimen). The last infant delivery date was 21 March 2022. We identified the first documented SARS-CoV-2 positive test date as the time of infection. Pregnant women with a positive SARS-CoV-2 test before or after pregnancy were excluded. We did not identify mothers with repeated positive tests during pregnancy. However, it is possible that some included cases may have had other asymptomatic infection episodes besides the one with the documented positive test. Maternal data included the date of the SARS-CoV-2 positive test, the date of delivery, the delivery method, medical comorbidities during pregnancy or labor, psychiatric diagnoses (depression, anxiety, or substance use) before or during pregnancy and any psychotropic medications during pregnancy. Infant data included sex, gestational age (in weeks) at time of delivery, age at every postnatal visit (in days), head circumference (in cm), and body weight (in kg) tracked longitudinally for one year following delivery. Infants receive regular routine visits during the first year of life. These visits include wellness checks for physical growth (i.e., body weight, head circumference) and development milestones. Subsequently, body weight and head circumference percentiles and z-scores were calculated for each measurement, considering age at time of measurement and infant sex using the World Health Organization (WHO) Child Growth Standards [36,37]. The trimester of maternal infection data was collected directly from the electronic health record (HER) and confirmed through the identification of the time interval between the date of first positive SARS-CoV-2 PCR test and the gestational age at delivery. All documented medical problems listed at the last visit were collected and categorized into the following 10 categories: (1) Feeding difficulties, (2) Speech and language delay, (3) vision impairment, (4) Seizures, (5) Developmental delay (delayed milestones, delayed speech, physiological developmental delay, deficit cognitive communication, or impairment of gross motor function), (6) Other neurological diagnoses (neurological spells, periodic limb movement disorder, encephalopathy, tremor, homonymous hemianopsia, cerebral palsy, microcephalus, neurogenic bladder, stereotyped movement disorder), (7) Cardiac defects, (8) Renal defects, (9) Spinal dysraphism, and (10) Hip dysplasia.

### 2.2. Statistical Analysis

Continuous data was displayed as mean and standard deviation (SD) and analyzed using General Linear Models; categorical data was displayed as counts (n) and percentages (%) and analyzed using chi-squared or Fisher’s Exact Test. Maternal data were grouped based on fetal sex and trimester of infection, and statistically significant differences between groups were planned to be included as covariates in the fetal outcomes analysis. Linear Mixed Models were used to analyze body weight and head circumference growth trajectories by sex and trimester and were computed with PROC MIXED. Mixed models were estimated with maximum likelihood, which can handle missing data in repeated measures designs [38,39], and used a variance components covariance matrix. The models included a random intercept per individual. Model parameters presented are unstandardized coefficients (B), *p*-values (*p*), and 95% confidence intervals (95% CI). Infant weight and head circumference percentile * sex trajectory models were as follows: Model 0 was an unadjusted model; Model 1 included clinically ascertained presence of developmental delay (yes/no); Model 2 included developmental delay, maternal age (in years), and maternal BMI (kg/m^2^). Infant weight and head circumference percentile * trimester exposure trajectory models were as follows: Model 0: unadjusted, Model 1: adjusted for comorbid anxiety and epidural anesthesia, Model 2: adjusted for comorbid anxiety, epidural anesthesia, maternal age (in years), and BMI (kg/m^2^). We evaluated alternative model specifications, including different random effects structures (random slope only, both random intercept and slope) and alternative covariance structures (unstructured, compound symmetry, autoregressive), using Akaike Information Criterion (AIC) and Bayesian Information Criterion (BIC). The random intercept model with variance components covariance structure demonstrated optimal fit with the lowest AIC and BIC values, indicating the best balance between model fit and parsimony. Trajectories of statistically significant interactions (age * sex or age * Trimester) were illustrated using LSMeans of the unadjusted model, with data points imported into GraphPad 10.1.2 (GraphPad Software, Inc., La Jolla, CA, USA). All data were analyzed using SAS 9.4 (SAS Institute; Cary, NC, USA). Statistical significance was denoted at *p* < 0.05 (two-tailed).

## 3. Results

### 3.1. Maternal Pregnancy and Delivery Data

The data for a total of 701 mothers and singleton neonates were collected and consisted of 357 (51%) male and 344 (49%) female neonates. The maternal SARS-CoV-2 positive test date was identified in the first, second and third trimesters in 160 (22.8%), 183 (26.1%) and 358 (51%) of cases, respectively. Maternal data were grouped based on infant sex and trimester of infection and were used to compare age, BMI at time of delivery, obstetrical history (number of previous pregnancies, full-term and preterm births, abortions, and living children), comorbidities during pregnancy, and delivery data. No significant differences were observed between the groups except in the prevalence of comorbid anxiety and in the use of epidural anesthesia during delivery. Both of these variables showed significant differences between trimester groups. Anxiety was reported at 33.1%, 26.2%, and 19.6% (*p* = 0.003) and epidural anesthesia was used in 72.5%, 60.1%, and 63.7%, (*p* = 0.047) of mothers who tested positive for SARS-CoV-2 in the 1st, 2nd, and 3rd trimesters, respectively (Table 1). The presence of comorbid anxiety or epidural anesthesia during delivery was used as a covariate in Model 2.

### 3.2. The Prevalence of Neurodevelopmental Disorders and Other Neurological Diagnoses Based on Sex and Trimester of Exposure

In the documented diagnoses reported during the first year of life, we observed that a higher percentage of male infants, compared to females, have neurodevelopmental diagnoses (10.9% vs. 5.2%, *p* = 0.008) and infants exposed to maternal SARS-CoV-2 infection in the second or third trimesters have a higher percentage of other neurological diagnoses compared to those exposed in the first trimester (1st vs. 2nd vs. 3rd TM: 0% vs. 0.9% vs. 0.7%, respectively, *p* = 0.037).

### 3.3. Differences in Infantile Body Weight and Head Circumference Percentile Growth Trajectories Based on Sex and Trimester of Exposure

More females, compared to males had at least one recorded BW and HC below the 10th percentile at some point during the first year of life [BW < 10th percentiles in females vs. males: 37.6% (129/244) vs. 24.1% (86/357), *p* = 0.0002 and HC < 10th percentiles in females vs. males: 34.5% (119/344) vs. 22.2% (79/357), *p* = 0.0003].

**Figure 1:**
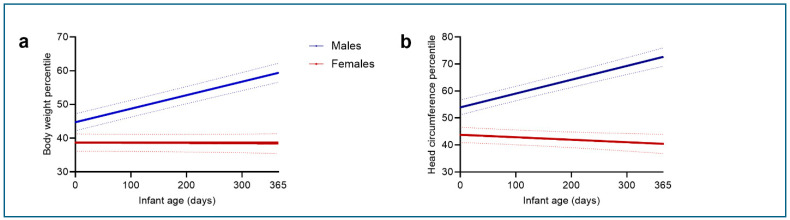
Growth percentile trajectories of (**a**) Body weight [B = −0.04, *p* < 0.0001, 95% Confidence Interval CI = −0.05 to −0.03] and (**b**) head circumference [B = −0.06, *p* < 0.0001, 95% Confidence Interval CI = −0.07 to −0.05] in male (blue) and female (red) infants over the first year of life using least square (LS) means of the unadjusted model (Model 0). Solid lines represent least square mean fitted trajectories; dotted lines indicate the 95% confidence intervals.

Female infants, compared to males, had significantly lower body weight and head circumference percentile growth trajectories over the first year of life (Figure 1a,b and Table 2). This sex difference in growth percentile trajectories was evident (*p* < 0.0001) in the uncorrected model (Model 0), and remained significant after adjusting for developmental delay (Model 1) and for developmental delay, maternal age and BMI (Model 2). This sex difference in body weight and head circumference growth percentiles was also detected in raw and z-score data (Supplemental Tables S1 and S2).

**Figure 2:**
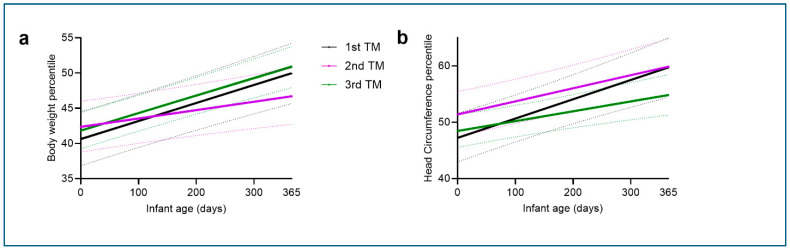
(**a**) Body weight [age * second trimester: B = −0.01, *p* = 0.006, 95% Confidence Interval CI = −0.02 to −0.003] and (**b**) head circumference [age * third trimester: B = −0.02, *p* = 0.02, 95% Confidence Interval CI = −0.03 to −0.002] percentile growth trajectories in first (black), second (purple) and third (green) trimester-exposed infants over the first year of life using least square (LS) means of the unadjusted model (Model 0). Solid lines represent least square mean fitted trajectories; dotted lines indicate the 95% confidence intervals.

Moreover, infants exposed to maternal SARS-CoV-2 infection in the second trimester had a significantly lower body weight percentile growth trajectory (Table 3 and Figure 2a), while infants exposed to maternal SARS-CoV-2 infection in the third trimester had a significantly lower head circumference percentile growth trajectory (Table 3 and Figure 2b). Again, these trimester differences were evident in the uncorrected model (Model 0) and remained significant after adjusting for comorbid anxiety and epidural anesthesia (Model 1) and for comorbid anxiety, epidural anesthesia, maternal age, and BMI (Model 2). The trimester effect was also significant in raw body weight (all models) and head circumference data (Model 1 only, Supplemental Table S3) and also for head circumference z-score (Models 1 and 2), but not body weight z-score data (Supplemental Table S4).

## 4. Discussion

In this study, we detected a higher prevalence of neurodevelopmental delay in male infants born to asymptomatic and mildly symptomatic mothers who tested positive for SARS-CoV-2 during pregnancy than in females. However, females had lower body weight and head circumference growth trajectories during the first year of life.

In addition, infants exposed to maternal SARS-CoV-2 infection during the second or third trimesters, compared to the first trimester, had a higher prevalence of neurological disorders, lower body weight and head circumference growth trajectories, respectively. The trimester-specific statistical differences in growth trajectory percentiles per day, despite seemingly small differences with uncertain clinical relevance, but still stresses the importance of follow-up of SARS-CoV-2 in utero exposed infants with particular attention to fetal sex and timing of exposure.

About 8% of infants in our cohort had a developmental delay diagnosis. These results are consistent with the prevalence of developmental delay in the general public before the pandemic [40,41,42] and with intrauterine exposure to SARS-CoV-2 [7,23]. However, the prevalence of developmental delay in our cohort is lower than the 10% rate reported in Ayad et al.’s study on 298 exposed infants [22], and the 12% rate reported in Fajardo-Martinez et al.’s study on 172 exposed children [10], or the 13.5% rate in Wu et al.’s study on 57 exposed children [9]. Studies that included control groups, such as the pan-Canadian Pregnancy During the COVID-19 Pandemic longitudinal study on 96 SARS-CoV-2-exposed and 800 unexposed children [14] and the study by Shuffrey et al. on 114 SARS-CoV-2-exposed and 141 unexposed infants, found no association between intrauterine exposure to SARS-CoV-2 and any neurodevelopmental outcomes between ages 6 and 24 months [13]. We did not include a SARS-CoV-2-negative control group in our study because the absence of symptoms or the identification of a negative PCR test at one time point is not enough to rule out infection. Many SARS-CoV-2 infections during pregnancy remain clinically silent [43], and the sensitivity of PCR varies with the timing of testing relative to viral exposure [44,45,46,47]. Instead, we examine the trimester effect, and our results suggest that the timing of in utero exposure to maternal SARS-CoV-2 infection may be associated with a significant effect on the rate of other neurological diagnoses (i.e., neurological spells, periodic limb movement disorder, encephalopathy, tremor, homonymous hemianopsia, cerebral palsy, microcephalus, neurogenic bladder, and stereotyped movement disorder) and on the growth trajectories later in life. Infants exposed in the second or third trimesters had higher rates of other neurological diagnoses, with a lower body weight growth trajectory in infants exposed in the second trimester and a lower head circumference growth trajectory in those exposed in the third trimester. Ayad et al. reported that the risk of developmental delays was higher in infants exposed in the first or second trimesters, compared to the third trimester [22], while Edlow et al. found that thirdtrimester infection was associated with a higher rate of neurodevelopmental diagnoses [23]. In contrast, the prospective ASPIRE (Assessing the Safety of Pregnancy in the Coronavirus Pandemic) study, which included 198 exposed and 1559 unexposed children at 12 months of age, did not find differences in the prevalence of developmental delay based on the trimester of infection [25].

The timing of in utero viral infection may affect fetal development differentially. One study reported that in utero exposure to the influenza virus during the 1918 pandemic increased the risk for specific organ injury depending on the month of gestation. Specifically, those born in May 1919 (who were conceived in August and in their 2nd month of gestation during the pandemic peak) had a higher diabetes risk than those exposed during other months of gestation [48]. According to Baker’s hypothesis, interruptions in normal in utero development can have lasting health effects specific to the organ system developing during a given trimester [49]. As such, we could envision more neurocognitive effects for children born to mothers infected in the third trimester because brain growth increases rapidly during that gestational period [50,51]. One brain imaging study quantified global and regional in vivo brain volumes using fetal magnetic resonance imaging (MRI) in a cohort of 64 healthy fetuses between 25 and 36 weeks of gestation. The study found that the cerebellum quadruples its volume, while the cerebral hemispheres double their volume during the third trimester [50]. Other studies provide further evidence that the third trimester is a period of rapid increase in total brain volume [52], sulci and gyri formation [53], white matter fiber bundle development [54], and myelination [55], which is essential for interneuronal connectivity and ultimately cognitive development [56]. A recent resting state functional MRI study reported that third trimester maternal immune activation is associated with alterations in salience network functional connectivity strength in the offspring at 40–44 weeks postmenstrual age, and maternal IL-6 levels correlated positively with measures of cognitive development at 14 months postmenstrual age [57]. Therefore, longitudinal imaging studies are needed to investigate the potential long-term effects of intra-uterine exposure.

We have observed a significantly higher prevalence of developmental delay in male infants, as well as lower body weight and head circumference growth trajectories in female infants. Male infants are more vulnerable to developmental delays than females [40,41,42]. Giuliani et al. reported a lower neonatal weight and head circumference at birth in a cohort of 586 neonates born to women with a COVID-19 diagnosis [58]. Ockene et al. also found lower body mass index (BMI) z-score at birth, but a greater gain in BMI z-score over the first year of life in SARS-CoV-2-exposed infants (n = 149) compared to unexposed infants (n = 127) [18]. In contrast, Eligulashvili et al. reported no differences in weight or head circumference growth curves between exposed (n = 758) and unexposed (n = 9345) children during the first two years of life [20].

Our results suggest that SARS-CoV-2-exposed male and female infants have different vulnerabilities. While males had more neurodevelopmental delays, females had lower head circumference and body weight percentile growth trajectories. A study from Edlow’s group found reduced maternal SARS-CoV-2-specific antibody titers as well as reduced transplacental transfer of these antibodies in women carrying male fetuses compared to those with female fetuses [59]. Maternal SARS-CoV-2 impacts placental viral entry gene expression (transmembrane protease, serine 2: TMPRSS2) in a sex-specific way [31] and placentas from SARS-CoV-2-infected mothers show inflammatory, thrombotic, and vascular changes, which could be associated with fetal growth restrictions [reviewed in [60,61,62]]. However, the SARS-CoV-2 virus itself rarely crosses the placental barrier, and only 2.2% of exposed newborns tested positive at the time of delivery in one study [63]. SARS-CoV-2 spike protein, on the other hand, was detected within the villous placenta in SARS-CoV-2-positive pregnant women [64] and spike protein administration to rats during pregnancy showed sex-specific effects in offspring. Specifically, male offspring exposed to the spike protein in utero displayed a higher rate of impaired performance on autism-like behavioral paradigms associated with hippocampal and cerebellar gliosis, neuronal cell death, and significantly greater levels of lipid peroxidation, neuroinflammatory markers, and reduced brain-derived neurotrophic factor (BDNF) than the control group [33].

The lower head circumference percentile growth trajectory in exposed females in our study, may be concerning, given that it could, to some extent, predict cognitive function in later life. Jensen et al. examined the association between full-scale IQ (WASI) and head circumference [65] in 123 adolescents, using head circumference measurements obtained during the third trimester of gestation via Doppler ultrasound. Head circumference was positively associated with fullscale IQ [66]. Similar results were reported in a Swedish study of 69 amphetamine (and other substances) addicted mothers, where fetal head circumference correlated with school achievement at 14 years of age in Swedish language and in mathematics grades in boys [67]. Another study from South India (n = 505 full-term born children) showed that learning ability, long-term storage and retrieval assessed by the Atlantis test in the Kaufman Assessment Battery for children-second edition, 2004 [68] Atlantis score, increased by 0.1 SD per SD increase in newborn head circumference [69]. Another population-based study of 1576 men and women born in Hertfordshire, Sheffield or Preston between 1920 and 1943 reported that scores on an intelligence test were higher in people who had a large biparietal head diameter at birth [70]. In addition, data from the British Cohort Study (n = 11,244) showed that head growth was a significant predictor of cognition at age 10, and the effect of head size on cognition was greater than the effect of birth weight [71].

Our clinical observations of subtle differences in growth trajectories call for further research to investigate potential neurobiological underpinnings. While our study does not establish biological causality, one hypothesis to explain the reported results could be maternal immune activation (MIA), which occurs when the mother’s immune system is activated during pregnancy in response to an infection, inflammation, or other immune stressors, without direct infection of the fetus. MIA during pregnancy has been repeatedly reported to be crucial for the development of neurodevelopmental disorders. A murine model of MIA showed a sex-specific effect where male offspring displayed alterations in pro-inflammatory cytokine levels and synaptic gene expression in association with behavioral changes, which was not observed in female offspring [72]. Notably, another study of murine MIA revealed that males had more severe placental pathology, fetal brain hypoxia, and social and learning-related behavioral abnormalities, while females exhibited postnatal growth delay and elevated anxiety-related behavior [73]. Notably, Gee et al. reported that neonates of mothers with recent or ongoing SARS-CoV-2 infection at the time of birth had distinct immune dysregulation, with activation of multiple innate and adaptive immune cells in neonatal cord blood. Interestingly, they reported that some changes in fetal immunity tend to recover with a longer interval between maternal COVID-19 and birth [74]. Furthermore, Díaz-Pons et al. reported an association between maternal and cord blood cytokine levels and Neonatal Behavioral Assessment Scale (NBAS) performance, with trimester-specific maternal immune activation impact (SARS-CoV-2 exposed mothers n = 59 vs. unexposed mothers n = 48) [75]. Longitudinal studies are necessary to assess the long-term brain structural and functional consequences of in utero exposure to SARS-CoV-2 infection, as well as the use of murine models to investigate the underlying pathogenesis.

We did not examine the effect of vaccination on growth trajectories or neurodevelopmental outcomes. Several studies that documented the effect of intrauterine exposure to maternal SARS-CoV-2 infection during the first year of life either excluded vaccinated cases to reduce variability [18,19] or did not comment on the vaccination status [13,20,23,24]. Data from the ASPIRE study showed that 30.6% (471/1541) of infants born to vaccinated mothers screened abnormally for developmental delay at 12 months, compared to 28.2% (203/720) of infants born to unvaccinated mothers [76]. One recent preclinical study examined the effect of maternal COVID-19 vaccination (inactivated Vero Cell administered on day E14.5, corresponding to the 2nd trimester in humans) on offspring development. They found that pups of vaccinated dams, compared to controls, showed enhanced working memory and hippocampal neurogenesis at 1 month of age [77]. Further research is needed to study the long-term safety of intrauterine exposure to SARS-CoV-2 infection and COVID-19 vaccination during pregnancy”.

The strengths of this study include the large sample with confirmed intrauterine exposure timing and the detailed phenotyping for maternal demographics, comorbid medical and psychiatric conditions, and pregnancy and labor data, in addition to the use of Linear Mixed Models and replicating the results of growth percentiles using row and also z-score body weight and head circumference data. We presented the growth percentiles, and not the z-scores, to show the growth values relative to normal growth curves, and not as the standard deviation from the mean. The limitations include the retrospective nature of the study, the fact that neurodevelopmental diagnoses were made clinically during routine visits with no standard diagnostic tools, and the absence of information on maternal socioeconomic status, inflammatory, viral load or symptomatic severity data, placental status or vertical transmission, as very few infants had SARS-CoV-2 PCR testing, trimester assignment is based on the first positive PCR only, while reinfection or asymptomatic intervals cannot be ruled out, and the exposure timing may be misclassified or imprecise, and the absence of a SARS-CoV-2 negative or pre-pandemic control group. Parental socioeconomic status (SES) specifically could have a significant effect on maternal immune activation (MAI) and on fetal neurodevelopment [78,79]. Data from the Healthcare Cost and Utilization Project Nationwide Inpatient Sample (HCUP-NIS) study reported that women from the wealthiest median household income were less likely to have an intrauterine fetal death but more likely to deliver neonates with congenital anomalies [80]. In addition, parental SES is associated with brain volumetric changes later in life [81]. Lu et al. reported a significant association between SES and in vivo fetal brain growth and cerebral cortical development. A higher SES was associated with significantly decreased fetal cortical gray matter volume, sulcal depth and increased white matter volumes in different brain regions [82].

Given the number of statistical comparisons performed, there is an increased risk of Type I error. However, we did not apply a correction for multiple testing because our primary outcomes (body weight and head circumference) are physiologically related and not independent, our hypotheses were pre-specified rather than exploratory, and analyses of raw values, z-scores, and percentiles represent different transformations of the same measurements rather than independent tests. Additionally, applying conservative corrections in this context would increase Type II error risk, potentially obscuring clinically relevant associations. Results should be interpreted as preliminary associations requiring replication in independent cohorts.

Despite these limitations, our results provide preliminary evidence that in utero exposure to maternal SARS-CoV-2 infection could have small but statistically detectable differences in early neurodevelopmental diagnoses and growth trajectories, which may vary by fetal sex and timing of exposure. Given the retrospective design, modest effect sizes, potential residual confounding, and the lack of a standardized developmental assessment or a non-exposed control group, these results should be interpreted cautiously. Longitudinal mother/infant dyad studies are needed to investigate the potential consequences of maternal SARS-CoV-2 infection timing on fetal/child brain development and cognition.

## Figures and Tables

**Table 1:** Maternal and fetal data.

Maternal and Fetal Data	Total, (n = 701)	Mothers of Male Neonates (n = 357)	Mothers of Female Neonates (n = 344)	*p* Value	Trimester of Maternal SARS-CoV-2 Positive Test			*p* Value
					1st (n = 160)	2nd (n = 183)	3rd (n = 358)	
Maternal age at time of delivery (mean ± SD)	29.7 ± 5.4	29.6 ± 5.4	29.8 ± 5.4	0.82	29.3 ± 5.4	30.3 ± 5.3	29.6 ± 5.4	0.2
Maternal BMI at time of delivery (mean ± SD)	32.6 ± 6.8	32.6 ± 6.6	32.7 ± 6.9	0.78	32.9 ± 7.3	32.5 ± 6.5	32.6 ± 6.6	0.84
BMI < 30, n (%)	265 (37.8%)	131 (36.7%)	134 (39.0%)	0.5	62 (38.7%)	67 (36.6%)	136 (38.0%)	0.99
BMI 30–40, n (%)	338 (48.2%)	180 (50.4%)	158 (45.9%)		77 (48.1%)	90 (49.2%)	171 (47.8%)	
BMI > 40, n (%)	98 (14.0%)	46 (12.9%)	52 (15.1%)		21 (13.1%)	26 (14.2%)	51 (14.3%)	
Obstetrical history								
Number of previous pregnancies (mean ± SD)	2.8 ± 2	2.8 ± 1.8	2.9 ± 1.9	0.49	2.7 ± 1.8	2.9 ± 1.9	2.9 ± 1.8	0.61
1 pregnancy, n (%)	178 (25.4%)	88 (24.7%)	90 (26.2%)	0.87	46 (28.9%)	48 (26.2%)	84 (23.5%)	0.58
2–4 pregnancies, n (%)	422 (60.3%)	218 (61.2%)	204 (59.3%)		90 (56.6%)	106 (57.9%)	226 (63.1%)	
>5 pregnancies, n (%)	100 (14.3%)	50 (14.0%)	50 (14.5%)		23 (14.5%)	29 (15.9%)	48 (13.4%)	
Previous births, mean (SD)	1.4 (1.3%)	1.4 (1.4%)	1.5 (1.3%)	0.6	1.3 (1.2%)	1.4 (1.4%)	1.5 (1.4%)	0.29
0 births, n (%)	181 (25.9%)	96 (27.0%)	85 (24.7%)	0.75	44 (27.7%)	50 (27.3%)	87 (24.3%)	0.59
1 birth, n (%)	239 (34.1%)	124 (34.8%)	115 (33.4%)		59 (37.1%)	63 (34.4%)	117 (32.7%)	
2 births, n (%)	159 (22.7%)	79 (22.2%)	80 (23.3%)		35 (22.0%)	36 (19.7%)	88 (24.6%)	
>3 births, n (%)	121 (17.3%)	57 (16.0%)	64 (18.6%)		21 (13.2%)	34 (18.6%)	66 (18.4%)	
Number of previous preterm births (mean ± SD)	0.1 ± 0.4	0.1 ± 0.5	0.1 ± 0.4	0.11	0.1 ± 0.3	0.2 ± 0.5	0.1 ± 0.5	0.08
0 preterm births, n (%)	633 (91.2%)	317 (89.6%)	316 (92.9%)	0.053	149 (94.9%)	156 (87.2%)	328 (91.6%)	0.28
1 preterm birth, n (%)	44 (6.3%)	28 (7.9%)	16 (4.7%)		7 (4.5%)	18 (10.1%)	19 (5.3%)	
2 preterm births, n (%)	12 (1.7%)	4 (1.1%)	8 (2.4%)		1 (0.6%)	4 (2.2%)	7 (2.0%)	
3 preterm births, n (%)	4 (0.6%)	4 (1.1%)	0 (0%)		0 (0%)	3 (0.6%)	1 (0.8%)	
4 preterm births, n (%)	1 (0.1%)	1 (0.3%)	0 (0%)		0 (0%)	0 (0%)	1 (0.1%)	
Number of abortions (mean ± SD)	0.5 ± 0.9	0.5 ± 0.8	0.6 ± 1.0	0.7	0.6 ± 1.1	0.5 ± 0.9	0.5 ± 0.9	0.7
0 abortions, n (%)	450 (64.8%)	231 (65.3%)	219 (64.4%)	0.25	103 (14.8%)	113 (63.1%)	234 (65.4%)	0.91
1 abortion, n (%)	161 (23.2%)	84(23.7%)	77 (22.7%)		33 (21.0%)	43 (24.0%)	85 (23.7%)	
2 abortions, n (%)	60 (8.7%)	32 (9.0%)	28 (8.2%)		14 (8.9%)	16 (8.9%)	30 (8.4%)	
>3 abortions, n (%)	23 (3.3%)	7 (2.0%)	16 (4.7%)		7 (4.5%)	7 (3.9%)	9 (2.5%)	
Number of children (mean ± SD)	1.6 ± 1.4	1.6 ± 1.5	1.6 ± 1.4	0.7	1.4 ± 1.3	1.6 ± 1.5	1.6 ± 1.4	0.71
0 children, n (%)	159 (22.9%)	83 (23.5%)	76 (22.4%)	0.99	40 (25.5%)	42 (23.5%)	77 (21.5%)	0.56
1 child, n (%)	235 (33.9%)	120 (33.9%)	115 (33.8%)		60 (38.2%)	57 (31.8%)	118 (33.0%)	
2 children, n (%)	164 (23.6%)	82 (23.2%)	82 (24.1%)		34 (21.7%)	43 (24.0%)	87 (24.3%)	
>3 children, n (%)	136 (19.6%)	69 (19.5%)	67 (19.7%)		23 (14.7%)	37 (20.7%)	76 (21.2%)	
Medical or psychiatric comorbidities during pregnancy								
Any comorbidities during pregnancy, n (%)	527 (75.2%)	262 (73.4%)	265 (77.0%)	0.26	116 (72.5%)	140 (76.5%)	271 (75.7%)	0.66
Advanced maternal age, n (%)	102 (14.6%)	53 (14.9%)	49 (14.2%)	0.82	20 (12.5%)	30 (16.4%)	52 (14.5%)	0.59
Major Depressive Disorder (MDD), n (%)	161 (23.0%)	76 (21.3%)	85 (24.7%)	0.28	40 (25.0%)	41 (22.4%)	80 (22.4%)	0.78
-MDD onset during pregnancy, n (%)	145 (20.7%)	71 (19.9%)	74 (21.5%)	0.55	40 (25.0%)	41 (22.4%)	77 (21.5%)	0.68
-MDD medication during pregnancy, n (%)	159 (22.7%)	76 (21.3%)	83 (23.8%)	0.43	40 (25.0%)	41 (22.4%)	78 (21.8%)	0.88
Anxiety comorbidities, n (%)	171 (24.4%)	89 (24.5%)	82 (23.8%)	0.76	53 (33.1%)	48 (26.2%)	70 (19.6%)	0.003
Drug use, n (%)	41 (5.9%)	21 (5.9%)	20 (5.8%)	0.96	7 (1%)	7 (1%)	27 (3.9%)	0.14
Seizures, n (%)	15 (2.1%)	8 (2.2%)	7 (2.0%)	1	3 (1.9%)	5 (2.7%)	7 (2.0%)	0.36
Seizure medications, n (%)	7 (1.0%)	4 (1.1%)	3 (0.9%)	0.4	2 (1.3%)	3 (1.6%)	3 (0.8%)	0.81
Diabetes, n (%)	97 (13.8%)	53 (14.9%)	44 (12.8%)	0.43	16 (10.0%)	29 (15.9%)	52 (14.5%)	0.25
Anemia, n (%)	82 (11.7)	37 (10.4)	45 (13.0)	0.26	16 (10.0)	22 (12.0)	44 (12.3)	0.75
Asthma, n (%)	49 (7.0)	23 (6.4)	26 (7.6)	0.56	10 (6.3)	12 (6.6)	27 (7.5)	0.84
Hypertension, n (%)	88 (12.6%)	46 (12.9%)	42 (12.2%)	0.79	28 (17.5%)	21 (11.5%)	39 (10.9%)	0.097
Preeclampsia, n (%)	42 (6.0%)	24 (6.7%)	18 (5.2%)	0.4	8 (5.0%)	11 (6.0%)	23 (6.4%)	0.82
GBS Positive, n (%)	112 (16.0%)	64 (17.9%)	48 (14.0%)	0.15	24 (15.0%)	28 (15.3%)	60 (16.7%)	0.85
Delivery data								
Gestational age (weeks) (mean ± SD)	38.8 ± 1.5	38.81.5	38.8 ± 1.6	0.82	38.7 ± 1.4	38.7 ± 1.8	38.9 ± 1.5	0.47
Induced labor, n (%)	336 (47.9%)	175 (49%)	161 (46.8%)	0.56	79 (49.4%)	90 (49.2%)	167 (46.7%)	0.79
Instrumental delivery, n (%)	20 (2.9%)	11 (3.1%)	9 (2.6%)	0.82	4 (2.5%)	5 (2.7%)	11 (3.0%)	0.93
Vaginal delivery, n (%)	474 (67.6%)	248 (69.5%)	226 (65.7%)	0.29	113 (70.6%)	117 (63.9%)	244 (68.2%)	0.4
Epidural anesthesia, n (%)	454 (64.8%)	243 (68.1%)	211 (61.3%)	0.06	116 (72.5%)	110 (60.1%)	228 (63.7%)	0.047
Cesarean section, n (%)	206 (29.4%)	94 (26.3%)	112 (32.6%)	0.07	42 (26.3%)	62 (33.9%)	102 (28.5%)	0.26
-Due to maternal indication, n (%)	91 (13.0%)	43 (12.0%)	48 (14.0%)	0.45	18 (11.3%)	29 (15.9%)	44 (12.3%)	0.39
-Due to fetal indication, n (%)	78 (11.1%)	32 (8.9%)	46 (13.4%)	0.06	18 (11.3%)	17 (9.3%)	43 (12.0%)	0.63
Appropriate for gestational age, n (%)	612 (87.3%)	309 (86.6%)	303 (88.1%)	0.52	137 (85.6%)	162 (88.5%)	313 (87.4%)	0.85
Larger for gestational age, n (%)	61 (8.7%)	35 (9.8%)	26 (7.6%)		15 (9.4%)	16 (8.7%)	30 (8.4%)	
Smaller for gestational age, n (%)	28 (4.0%)	13 (3.6%)	15 (4.3%)		8 (5.0%)	5 (2.7%)	15 (4.2%)	
IUGR, n (%)	15 (2.1%)	5 (1.4%)	10 (2.9%)	0.2	2 (1.3%)	3 (1.6%)	10 (2.8%)	0.45
APGAR score at 1 min (mean ± SD)	7.8 ± 1.4	7.8 ± 1.3	7.8 ± 1.5	0.7	7.7 ± 1.5	7.7 ± 1.5	7.8 ± 1.3	0.5
APGAR score at 5 min (mean ± SD)	8.7 ± 0.9	8.7 ± 0.8	8.7 ± 1.0	0.9	8.7 ± 0.8	8.6 ± 1.1	8.8 ± 0.9	0.4
**Infant diagnoses during the first year of life**	**All**	**Males**	**Females**	***p* value**	**Exposed in the 1st trimester**	**Exposed in the 2nd trimester**	**Exposed in the 3rd trimester**	***p* value**
Feeding difficulties, n (%)	4 (0.6%)	4 (1.1%)	0 (0%)	0.12	0 (0%)	1 (0.6%)	3 (0.8%)	0.8
Speech or language delay, n (%)	4 (0.6%)	3 (0.84%)	1 (0.3%)	0.62	0 (0%)	2 (1.1%)	2 (0.6%)	0.56
Vision impairment, n (%)	17 (2.4%)	9 (2.5%)	8 (2.3%)	0.86	4 (2.5%)	4 (2.2%)	9 (2.5%)	0.99
Seizures, n (%)	7 (1%)	4 (1.1%)	3 (0.9%)	0.99	1 (0.6%)	3 (1.6%)	3 (0.8%)	0.6
Developmental delay *, n (%)	57 (8.1%)	39 (10.9%)	18 (5.2%)	0.008	13 (8.1%)	18 (9.8%)	26 (7.3%)	0.56
Other neurological diagnoses **, n (%)	11 (1.6)	5 (1.4)	6 (1.7)	0.77	0 (0)	6 (0.9)	5 (0.7)	0.037
Cardiac defect, n (%)	10 (1.4%)	3 (0.84%)	7 (2.03%)	0.2	0 (0%)	4 (2.2%)	6 (1.7%)	0.2
Renal defects, n (%)	5 (0.7%)	3 (0.8%)	2 (0.6%)	0.99	0 (0%)	1 (0.6%)	4 (1.1%)	0.5
Spinal dysraphism, n (%)	2 (0.3%)	1 (0.3%)	1 (0.3%)	0.99	1 (0.1%)	1 (0.6%)	0 (0%)	0.2
Hip dysplasia, n (%)	2 (0.3%)	0 (0%)	2 (0.6%)	0.24	1 (0.6%)	0 (0%)	1 (0.3%)	0.5

notes: * Developmental delay included: delayed milestones, delayed speech, physiological developmental delay, deficit cognitive communication, impairment of gross motor function. ** Other Neurological diagnoses include: neurological spells, periodic limb movement disorder, encephalopathy, tremor, homonymous hemianopsia, cerebral palsy, microcephalus, neurogenic bladder, and stereotyped movement disorder.

**Table 2:** Body weight and head circumference percentile * sex trajectory Linear Mixed Models analyses.

Body Weight Percentile * Sex Trajectory Analyses	Body Weight Percentile * Sex Models											
	Model 0: Unadjusted				Model 1: Adjusted for Developmental Delay				Model 2: Adjusted for Developmental Delay, Maternal Age, and BMI			
	B	*p*	95% CI		B	*p*	95% CI		B	*p*	95% CI	
Infant Age (days)	0.04	<0.0001	0.04	0.04	0.04	<0.0001	0.04	0.04	0.04	<0.0001	0.04	0.04
Infant Female 2	−6.05	0.001	−9.65	−2.44	−5.86	0.0016	−9.49	−2.23	−6.13	0.0009	−9.74	−2.51
Infant Male 1	REF				REF				REF			
Infant Age * Infant Male 1	−0.04	<0.0001	−0.05	−0.03	−0.04	<0.0001	−0.05	−0.03	−0.04	<0.0001	−0.05	−0.03
Infant Age * Infant Female 2	REF				REF				REF			
Presence of Developmental Delay 1	--				3.01	0.37	−3.61	9.62	2.92	0.39	−3.67	9.51
No Presence of Developmental Delay 0	--				REF				REF			
Maternal Age (years)	--				--				−0.11	0.52	−0.44	0.22
Maternal BMI (kg/m^2^)	--				--				0.31	0.02	0.05	0.57
Head circumference percentile * sex trajectory analyses	Head circumference percentile * sex models											
	**Model 0: Unadjusted**				**Model 1: Adjusted for developmental delay**				**Model 2: Adjusted for developmental delay, maternal age, and BMI**			
	**B**	***p***	**95% CI**		**B**	***p***	**95% CI**		**B**	***p***	**95% CI**	
Infant Age (days)	0.05	<0.0001	0.04	0.06	0.05	<0.0001	0.04	0.06	0.05	<0.0001	0.04	0.06
Infant Female 2	−10.2	<0.0001	−14.17	−6.24	−10.11	<0.0001	−14.1	−6.12	−10.62	<0.0001	−14.59	−6.64
Infant Male 1	REF				REF				REF			
Infant Age * Infant Female 2	−0.06	<0.0001	−0.07	−0.05	−0.06	<0.0001	−0.07	−0.05	−0.06	<0.0001	−0.07	−0.05
Infant Age * Infant Male 1	REF				REF				REF			
Presence of Developmental Delay 1	--				1.5	0.68	−5.59	8.58	1.13	0.75	−5.92	8.18
No Presence of Developmental Delay 0	--				REF				REF			
Maternal Age (years)	--				--				0.11	0.55	−0.25	0.47
Maternal BMI (kg/m^2^)	--				--				0.26	0.07	−0.02	0.54

**Table 3:** Body weight and head circumference percentile * trimester of exposure trajectory Linear Mixed Models analyses.

Body Weight Percentile * Trimester of Exposure Trajectory Analyses	Body Weight Percentile * Trimester of Exposure Models											
	Model 0: Unadjusted				Model 1: Adjusted for Comorbid Anxiety and Epidural Anesthesia				Model 2: Adjusted for Comorbid Anxiety, Epidural Anesthesia, Maternal Age, and BMI			
	B	*p*	95% CI		B	*p*	95% CI		B	*p*	95% CI	
Infant Age (days)	0.03	<0.0001	0.02	0.03	0.03	<0.0001	0.02	0.03	0.03	<0.0001	0.02	0.03
Trimester 2	1.72	0.52	−3.54	6.97	2.12	0.43	−3.15	7.38	2.56	0.34	−2.7	7.82
Trimester 3	1.16	0.62	−3.45	5.78	1.35	0.57	−3.3	5.99	1.77	0.46	−2.88	6.41
Trimester 1	REF				REF				REF			
Infant Age * Trimester 2	−0.01	0.006	−0.02	−3.89 × 10^−3^	−0.01	0.006	−0.02	−3.89 × 10^−3^	−0.01	0.0065	−0.02	−3.77 × 10^−3^
Infant Age * Trimester 3	−7 × 10^−4^	0.87	−9.36 × 10^−3^	7.96 × 10^−3^	−6.7 × 10^−4^	0.88	−9.33 × 10^−3^	7.99 × 10^−3^	−5.70 × 10^−4^	0.9	−9.25 × 10^−3^	8.11 × 10^−3^
Infant Age * Trimester 1	REF				REF				REF			
Presence of Psychiatric Comorbidity (anxiety) 1	--				−0.98	0.65	−5.21	3.25	−1.14	0.6	−5.37	3.08
No Presence of Psychiatric Comorbidity (anxiety) 0	--				REF				REF			
Epidural anesthesia Use 1	--				3.72	0.06	−0.09	7.53	3.7	0.06	−0.12	7.52
No Epidural anesthesia Use 0	--				REF				REF			
Maternal Age (years)	--				--				−0.08	0.66	−0.42	0.26
Maternal BMI (kg/m^2^)	--				--				0.32	0.02	0.05	0.59
Head circumference percentile * trimester of exposure trajectory analyses	Head circumference percentile * trimester of exposure models											
	**Model 0: Unadjusted**				**Model 1: Adjusted for comorbid anxiety and epidural anesthesia**				**Model 2: Adjusted for comorbid anxiety, epidural anesthesia, maternal age, and BMI**			
	**B**	***p***	**95% CI**		**B**	***p***	**95% CI**		**B**	***p***	**95% CI**	
Infant Age (days)	0.03	<0.0001	0.02	0.05	0.03	<0.0001	0.02	0.05	0.03	<0.0001	0.02	0.05
Trimester 2	4.15	0.17	−1.76	10.05	4.55	0.13	−1.37	10.48	4.95	0.1	−0.97	10.88
Trimester 3	1.18	0.66	−4.02	6.37	1.44	0.59	−3.79	6.67	2.1	0.43	−3.13	7.33
Trimester 1	REF				REF				REF			
Infant Age * Trimester 2	−0.01	0.18	−0.03	5.11 × 10^−3^	−0.01	0.18	−0.03	5.09 × 10^−3^	−0.01	0.17	−0.03	4.75 × 10^−3^
Infant Age * Trimester 3	−0.02	0.02	−0.03	−2.25 × 10^−3^	−0.02	0.02	−0.03	−2.23 × 10^−3^	−0.02	0.02	−0.03	−2.64 × 10^−3^
Infant Age * Trimester 1	REF				REF				REF			
Presence of Psychiatric Comorbidity (anxiety) 1	--				−0.15	0.95	−4.81	4.52	−0.11	0.96	−4.76	4.55
No Presence of Psychiatric Comorbidity (anxiety) 0	--				REF				REF			
Epidural anesthesia Use 1	--				3.35	0.12	−0.87	7.56	3.64	0.09	−0.59	7.86
No Epidural anesthesia Use 0	--				REF				REF			
Maternal Age (years)	--				--				0.12	0.52	−0.25	0.5
Maternal BMI (kg/m^2^)	--				--				0.27	0.08	−0.03	0.56

## Data Availability

The patient data used in this study is unsuitable for publication due to confidential information.

## Supplementary Materials

The following are available online at https://www.mdpi.com/article/10.3390/jcm15020600/s1, Table S1: Raw body weight and head circumference * sex trajectory Linear Mixed Models analyses, Table S2: Z-score body weight and head circumference * sex trajectory Linear Mixed Models analyses, Table S3: Raw body weight and head circumference percentile * trimester of exposure trajectory Linear Mixed Models analyses, Table S4: Z-score body weight and head circumference percentile * trimester of exposure trajectory Linear Mixed Models analyses.
