# Supplementary material for: The Timing of Intrauterine Exposure to Maternal SARS-CoV-2 Infection Impacts Neurodevelopment and Growth Trajectories During the First Year of Life
Source: J Clin Med. 2026 Jan 12;15(2):600. doi: 10.3390/jcm15020600 (PMC12841638; doi:10.3390/jcm15020600)
Supplement: Supplementary file 1 [file jcm-15-00600-s001.zip › jcm-4043523-supplementary.pdf]

Journal of Clinical Medicine

MDPI

Table S1: Raw body weight and head circumference*sex trajectory Linear Mixed Models analyses.

Raw body weight (Kg)*sex trajectory analyses (Linear Mixed Models)	Raw body weight (Kg)*sex models											
	Model 0: Unadjusted				Model 1: Adjusted for developmental delay				Model 2: Adjusted for developmental delay, maternal age, and BMI			
	B	P	95% CI		B	P	95% CI		B	P	95% CI	
Infant Age (days)	0.02	<.0001	0.02	0.02	0.02	<.0001	0.02	0.02	0.02	<.0001	0.02	0.02
Infant Female 2	-0.15	0.0031	-0.24	-0.05	-0.14	0.0047	-0.24	-0.04	-0.15	0.0031	-0.24	-0.05
Infant Male 1	REF				REF				REF			
Infant Age*Infant Female 2	-1.50E-03	<.0001	-1.75E-03	-1.25E-03	-1.50E-03	<.0001	-1.75E-03	-1.25E-03	-1.50E-03	<.0001	-1.75E-03	-1.24E-03
Infant Age*Infant Male 1	REF				REF				REF			
Presence of Developmental Delay 1	--				0.1	0.27	-0.08	0.27	0.09	0.29	-0.08	0.27
No Presence of Developmental Delay 0	--				--				REF			
Maternal Age (years)	--				--				-3.43E-03	0.44	-0.01	5.37E-03
Maternal BMI (kg/m2)	--				--				7.25E-03	0.04	2.91E-04	0.01

Raw head circumference (cm)*sex trajectory analyses (Linear Mixed Models)	Raw head circumference (cm)*sex models											
	Model 0: Unadjusted				Model 1: Adjusted for developmental delay				Model 2: Adjusted for developmental delay, maternal age, and BMI			
	B	P	95% CI		B	P	95% CI		B	P	95% CI	
Infant Age (days)	0.04	<.0001	0.04	0.05	0.04	<.0001	0.04	0.05	0.04	<.0001	0.04	0.05
Infant Female 2	-0.59	0.0361	-1.15	-0.04	-0.61	0.03	-1.17	-0.05	-0.63	0.0267	-1.19	-0.07
Infant Male 1	REF				REF				REF			
Infant Age*Infant Female 2	-5.31E-03	0.0083	-9.25E-03	-1.37E-03	-5.33E-03	0.0081	-9.27E-03	-1.38E-03	-5.22E-03	0.0096	-9.17E-03	-1.27E-03
Infant Age*Infant Male 1	REF				REF				REF			
Presence of Developmental Delay 1					-0.34	0.37	-1.07	0.39	-0.36	0.34	-1.09	0.38
No Presence of Developmental Delay 0					REF				REF			
Maternal Age (years)									0.01	0.56	-0.03	0.05

Maternal BMI (kg/m2)									6.94E-03	0.65	-0.02	0.04

Table S2: Z-score body weight and head circumference*sex trajectory Linear Mixed Models analyses.

**Body weight (z-score)*sex trajectory analyses (Linear Mixed Models)**	**body weight (z-score)*sex models**											
	**Model 0: Unadjusted**				**Model 1: Adjusted for developmental delay**				**Model 2: Adjusted for developmental delay, maternal age, and BMI**			
	**B**	**P**	**95% CI**		**B**	**P**	**95% CI**		**B**	**P**	**95% CI**	
Infant Age (days)	1.79E-03	<.0001	1.17E-03	2.41E-03	1.79E-03	<.0001	1.62E-03	1.96E-03	1.79E-03	<.0001	1.61E-03	1.96E-03
Infant Female 2	-0.23	0.0073	-0.39	-0.06	-0.23	0.0018	-0.37	-0.08	-0.24	0.0009	-0.38	-0.1
Infant Male 1	REF				REF				REF			
Infant Age*Infant Female 2	-1.43E-03	0.0011	-2.28E-03	-5.70E-04	-1.43E-03	<.0001	-1.68E-03	-1.17E-03	-1.43E-03	<.0001	-1.68E-03	-1.17E-03
Infant Age*Infant Male 1	REF				REF				REF			
Presence of Developmental Delay 1					0.03	0.81	-0.23	0.29	0.02	0.85	-0.23	0.28
No Presence of Developmental Delay 0	--				REF				REF			
Maternal Age (years)	--				--				-2.31E-03	0.73	-0.02	0.01
Maternal BMI (kg/m2)	--				--				0.01	0.02	2.07E-03	0.02

**Head circumference (z-score)*sex trajectory analyses (Linear Mixed Models)**	**Head circumference (z-score)*sex models**											
	**Model 0: Unadjusted**				**Model 1: Adjusted for developmental delay**				**Model 2: Adjusted for developmental delay, maternal age, and BMI**			
	**B**	**P**	**95% CI**		**B**	**P**	**95% CI**		**B**	**P**	**95% CI**	
Infant Age (days)	5.63E-03	0.07	-5.30E-04	0.01	5.65E-03	<.0001	3.55E-03	7.76E-03	5.59E-03	<.0001	3.48E-03	7.70E-03
Infant Female 2	-0.33	0.2	-0.84	0.18	-0.35	0.12	-0.78	0.09	-0.36	0.1	-0.8	0.07
Infant Male 1	REF				REF				REF			
Infant Age*Infant Female 2	-5.06E-03	0.11	-0.01	1.22E-03	-5.07E-03	0.0013	-8.16E-03	-1.99E-03	-4.97E-03	0.0016	-8.06E-03	-1.89E-03
Infant Age*Infant Male 1	REF				REF				REF			

Presence of Developmental Delay 1					-0.32	0.28	-0.89	0.26	-0.33	0.26	-0.91	0.24
No Presence of Developmental Delay 0					REF				REF			
Maternal Age (years)									9.83E-03	0.52	-0.02	0.04
Maternal BMI (kg/m2)									7.79E-03	0.51	-0.01545	0.03

Table S3: Raw body weight and head circumference percentile*trimester of exposure trajectory Lin-ear Mixed Models analyses.

Raw body weight (Kg)*trimester of exposure trajectory analyses (Linear Mixed Models)	Raw body weight (Kg)*trimester of exposure models											
	Model 0: Unadjusted				Model 1: Adjusted for comorbid anxiety and epidural anesthesia				Model 2: Adjusted for comorbid anxiety, epidural anesthesia, maternal age, and BMI			
	B	P	95% CI		B	P	95% CI		B	P	95% CI	
Infant Age (days)	0.02	<.0001	0.02	0.02	0.02	<.0001	0.02	0.02	0.02	<.0001	0.02	0.02
Trimester 2	-8.06E-03	0.91	-0.15	0.13	7.29E-03	0.92	-0.13	0.15	0.01	0.87	-0.13	0.15
Trimester 3	8.79E-04	0.99	-0.12	0.13	9.34E-03	0.88	-0.12	0.13	0.02	0.8	-0.11	0.14
Trimester 1	REF				REF				REF			
Infant Age*Trimester 2	-4.20E-04	0.02	-7.70E-04	-6.00E-05	-4.20E-04	0.02	-7.70E-04	-6.00E-05	-4.20E-04	0.02	-7.70E-04	-6.00E-05
Infant Age*Trimester 3	6.00E-05	0.71	-2.60E-04	3.80E-04	6.20E-05	0.7	-2.60E-04	3.81E-04	5.80E-05	0.72	-2.60E-04	3.78E-04
Infant Age*Trimester 1	REF				REF				REF			
Presence of Psychiatric Comorbidity (anxiety) 1	--				-0.02	0.7	-0.13	0.09	-0.03	0.6	-0.14	0.08
No Presence of Psychiatric Comorbidity (anxiety) 0	--				REF				REF			
Epidural anesthesia Use 1	--				0.14	0.0083	0.04	0.24	0.14	0.007	0.04	0.24
No Epidural anesthesia Use 0	--				REF				REF			
Maternal Age (years)	--				--				-1.76E-03	0.7	-0.01	7.31E-03

Maternal BMI (kg/m2)	--				--				7.59E-03	0.04	4.90E-04	0.01

**Raw head circumference (cm)*trimester of exposure trajectory analyses (Linear Mixed Models)**	**Raw head circumference (cm)*trimester of exposure models**											
	**Model 0: Unadjusted**				**Model 1: Adjusted for comorbid anxiety and epidural anesthesia**				**Model 2: Adjusted for comorbid anxiety, epidural anesthesia, maternal age, and BMI**			
	**B**	**P**	**95% CI**		**B**	**P**	**95% CI**		**B**	**P**	**95% CI**	
Infant Age (days)	0.04	<.0001	0.04	0.04	0.07	<.0001	0.06	0.09	0.04	<.0001	0.04	0.04
Trimester 2	-0.69	0.09	-1.48	0.1	-0.04	0.91	-0.79	0.71	-0.64	0.11	-1.44	0.15
Trimester 3	0.08	0.83	-0.63	0.78	0.66	0.05	-1.75E-03	1.32	0.15	0.68	-0.56	0.86
Trimester 1	REF				REF				REF			
Infant Age*Trimester 2	9.37E-03	0.0009	3.82E-03	0.01	-6.20E-04	0.95	-0.02	0.02	9.31E-03	0.001	3.75E-03	0.01
Infant Age*Trimester 3	-1.30E-04	0.96	-5.05E-03	4.78E-03	-0.04	<.0001	-0.06	-0.03	-1.90E-04	0.94	-5.12E-03	4.73E-03
Infant Age*Trimester 1	REF				REF				REF			
Presence of Psychiatric Comorbidity (anxiety) 1	--				0.15	0.58	-0.38	0.69	0.14	0.56	-0.33	0.61
No Presence of Psychiatric Comorbidity (anxiety) 0	--				REF				REF			
Epidural anesthesia Use 1	--				0.24	0.35	-0.26	0.74	0.41	0.07	-0.03	0.85
No Epidural anesthesia Use 0	--				REF				REF			
Maternal Age (years)	--				--				0.02	0.43	-0.02	0.05
Maternal BMI (kg/m2)	--				--				8.40E-03	0.58	-0.02	0.04

Table S4: Z-score body weight and head circumference percentile*trimester of exposure trajectory Linear Mixed Models analyses.

**Body weight (z-score)*trimester of exposure trajectory analyses (Linear Mixed Models)**	**Body weight (z-score)*trimester of exposure models**											
	**Model 0: Unadjusted**				**Model 1: Adjusted for comorbid anxiety and epidural anesthesia**				**Model 2: Adjusted for comorbid anxiety, epidural anesthesia, maternal age, and BMI**			
	**B**	**P**	**95% CI**		**B**	**P**	**95% CI**		**B**	**P**	**95% CI**	
Infant Age (days)	1.26E-03	<.0001	9.92E-04	1.52E-03	1.26E-03	<.0001	9.92E-04	1.52E-03	1.26E-03	<.0001	9.90E-04	1.52E-03

Trimester 2	0.02	0.87	-0.19	0.22	0.04	0.72	-0.17	0.24	0.05	0.62	-0.15	0.26
Trimester 3	0.05	0.62	-0.13	0.23	0.05	0.57	-0.13	0.23	0.07	0.45	-0.11	0.25
Trimester 1	REF				REF				REF			
Infant Age*Trimester 2	-1.80E-04	0.33	-5.40E-04	1.80E-04	-1.80E-04	0.33	-5.40E-04	1.80E-04	-1.80E-04	0.33	-5.40E-04	1.80E-04
Infant Age*Trimester 3	-1.50E-04	0.36	-4.80E-04	1.71E-04	-1.50E-04	0.36	-4.70E-04	1.73E-04	-1.50E-04	0.36	-4.80E-04	1.73E-04
Infant Age*Trimester 1	REF				REF				REF			
Presence of Psychiatric Comorbidity (anxiety) 1	--				-0.07	0.41	-0.23	0.1	-0.08	0.36	-0.24	0.09
No Presence of Psychiatric Comorbidity (anxiety) 0	--				REF				REF			
Epidural anesthesia Use 1	--				0.19	0.01	0.04	0.34	0.2	0.0098	0.05	0.34
No Epidural anesthesia Use 0	--				REF				REF			
Maternal Age (years)	--				--				-8.40E-04	0.9	-0.01	0.01
Maternal BMI (kg/m2)	--				--				0.01	0.02	2.29E-03	0.02

**Head circumference (z-score)*trimester of exposure trajectory analyses (Linear Mixed Models)**	**Head circumference (z-score)*trimester of exposure models**											
	**Model 0: Unadjusted**				**Model 1: Adjusted for comorbid anxiety and epidural anesthesia**				**Model 2: Adjusted for comorbid anxiety, epidural anesthesia, maternal age, and BMI**			
	**B**	**P**	**95% CI**		**B**	**P**	**95% CI**		**B**	**P**	**95% CI**	
Infant Age (days)	1.67E-03	0.3	-1.49E-03	4.83E-03	1.67E-03	0.3	-1.49E-03	4.83E-03	1.70E-03	0.29	-1.46E-03	4.87E-03
Trimester 2	-0.54	0.09	-1.17	0.08	-0.51	0.11	-1.13	0.12	-0.5	0.12	-1.12	0.13
Trimester 3	0.1	0.71	-0.45	0.66	0.15	0.6	-0.41	0.7	0.16	0.56	-0.39	0.72
Trimester 1	REF				REF				REF			
Infant Age*Trimester 2	7.38E-03	0.0009	3.04E-03	0.01	7.37E-03	0.0009	3.03E-03	0.0117	7.30E-03	0.001	2.96E-03	0.01164
Infant Age*Trimester 3	-5.80E-04	0.77	-4.43E-03	3.26E-03	-5.80E-04	0.77	-4.42E-03	3.27E-03	-6.40E-04	0.74	-4.49E-03	3.20E-03
Infant Age*Trimester 1	REF				REF				REF			
Presence of Psychiatric Comorbidity (anxiety) 1	--				0.08	0.66	-0.29	0.45	0.09	0.62	-0.27	0.46

No Presence of Psychiatric Comorbidity (anxiety) 0	--	REF				REF			
Epidural anesthesia Use 1	--	0.32	0.06	-0.02	0.66	0.35	0.05	2.89E-03	0.69
No Epidural anesthesia Use 0	--	REF				REF			
Maternal Age (years)	--	--				0.01	0.4	-0.02	0.04
Maternal BMI (kg/m2)	--	--				8.90E-03	0.4567	-0.01	0.03
